# Where There Is No Internet: Delivering Health Information via the Blue Trunk Libraries

**DOI:** 10.1371/journal.pmed.0030077

**Published:** 2006-03-07

**Authors:** Pascal Mouhouelo, Auguste Okessi, Marie-Paule Kabore

## Abstract

There are many areas in the developing world that have neither computers nor a reliable electricity supply. In response to the need for printed health information, WHO librarians created the Blue Trunk Library Project.

## The Technology Gap

“Information access,” argued Packenham-Walsh and colleagues in the *BMJ*, “is the sine qua non of the professional development of all health workers—the most vital asset of any healthcare system” [ 1]. And the development of the Internet has brought hope that access to health information might one day become universal. Access to online databases gives users the opportunity to retrieve a wealth of relevant and up-to-date information. Every day, health-related books, research, and other articles are retrieved from the Internet, which has led to a worldwide information revolution ( Box 1).

However, the World Health Organization (WHO) is very much aware that there are many areas in the world where access to the Internet is not yet a reality. In developing countries, a large proportion of the population, including health professionals, has no or only poor access to the Internet. Even printed materials, such as up-to-date books, current periodicals, and newspapers, are scarce. In this situation, professionals are obliged to rely on the knowledge acquired during their original training to care for patients, to prevent disease, and to promote health.

In many regions, the health district centers are staffed by nurses, midwives, and community health workers who, having finished their basic studies, receive little in the way of continuing education, as libraries rarely exist at the district level or in regional hospitals. The distribution of CD-ROMs to developing countries is an important initiative, which has proven to be a valuable source of health information. For example, the health-related CD-ROMs from TALC (Teaching-aids At Low Cost, http://www.talcuk.org) [ 2] and those distributed by the WHO and the joint United Nations Programme for HIV/AIDS (UNAIDS) are much appreciated by their users. The CD-ROM is an important tool for information delivery in Africa because it does not take up a lot of space and shipping it is inexpensive.

Unfortunately, there are still many areas in the developing world that have neither computers nor a reliable electricity supply. Thus, in spite of the rapid development of information and communications technologies, the gap between “the haves and have-nots” continues to blight isolated areas (those outside a capital city). In these areas, the appropriate solution to information access is still printed material. In response to this need for printed health information, WHO librarians created the Blue Trunk Library (BTL) project.

## What Is the Blue Trunk Library?

The idea of creating the BTL collection stemmed from the conclusions of a joint survey conducted by the ministry of health and the WHO country office in Guinea to define the continuing education needs for health workers based in the health districts. The survey found that these workers needed to broaden their skills. Therefore, the WHO library was asked to compile a collection of appropriate books to suit the different education needs of health district workers at various professional levels. The WHO library in Geneva started the BTL project in 1998. Guinea was the first country to benefit from this prototype [ 3], which was then extended to other African countries before being taken up in developing countries in other parts of the world. There are English and French versions of the BTL.

**Figure 1:**
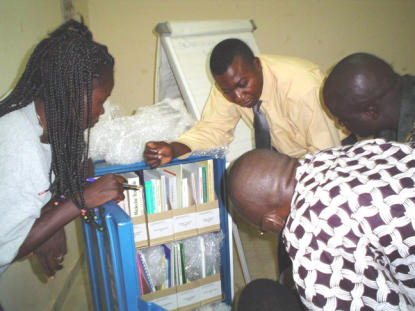
A Blue Trunk Library Training Session

The BTL is “a ready-to-use documentation module” [ 4] of about 150 WHO and non-WHO books and manuals fitted into a blue metal trunk ( Figure 1). The materials are arranged and filed in such a way that users can easily identify the ones that they need. Fourteen topics have been chosen using a basic classification code, e.g., General Medicine and Nursing (100), Community Health (110), and these codes are written on each filing box.

The content is selected by the WHO press unit and the WHO library in collaboration with a group of WHO health professionals. The BTL's composition varies according to the books available in each language. For example, *Médecine Tropicale* by Marc Gentilini [ 5] is selected for the French version of the BTL, while the *Oxford Handbook of Tropical Medicine* [ 6] is selected for the English version. Books dealing with specific diseases are removed or replaced according to the needs of the specific geographical area that is receiving the BTL.

**Figure 2:**
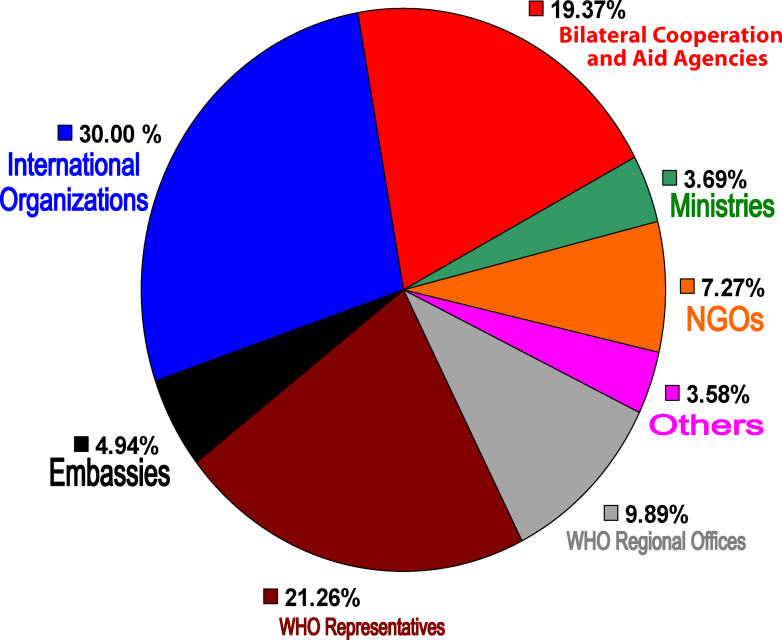
Types of Sponsorship for the Blue Trunk Libraries

Each BTL costs US$2,000, which includes training users but not the cost of shipping (it costs about US$717 to send a BTL to Lusaka, Zambia, and about US$620 to Yaoundé, Cameroon). The BTL project is funded by international and bilateral donors such as the United Nations Children's Fund (UNICEF), the United Nations Development Programme (UNDP), the WHO, the German Council for Sustainable Development (GTZ), the United States Agency for International Development (USAID); Belgian, Italian, French, German, and Irish embassies; and nongovernmental organizations such as Save the Children, Merlin, and Entre-Aide (see types of sponsorship in Figure 2). These donors and NGOs have funded BTLs in countries where they have development projects. Funders purchase a BTL directly from the BTL management team in Geneva (E-mail: bluetrunk@who.int).

In countries where at least 20 BTLs have been acquired, the ministry of health appoints a national coordinator, and assistants are selected from the health districts to attend a training session in the use of the BTL ( Figure 1). The BTL project supports this training, which is facilitated by WHO librarians and national health librarians.

## The African Experience

Health districts in the anglophone and francophone countries of Africa have been the major beneficiaries of the BTL. Training sessions have been organized in 14 countries, including Burundi, Guinea, Ethiopia, Republic of Congo, Mauritania, and Mali. Commitment to this project is shared both by donors (funding a BTL addresses the information needs of isolated rural Africa) and by the community health workers (who are given an opportunity to broaden their skills).

To date, the sub-Saharan countries have acquired about 850 BTLs out of the total of 1,488 distributed worldwide. The ambitious objective is to provide each health district with one BTL, but these districts are not the only health-related structures in need. For example, the WHO Regional Office library has received requests from nursing schools and NGOs in Zimbabwe, Burundi, Sierra Leone, and the Republic of Congo.

One of the problems the project is facing is how best to reach African lusophone countries. While it is reasonably easy to identify suitable materials in English and French, this is not the case for Portuguese learning materials. We tried to translate some of the BTL contents into Portuguese, but with little success because of the large number of books that needed to be translated. Collaboration with institutions in Brazil that generate health materials themselves is likely to be a more fruitful strategy. Portuguese-speaking countries should not be left out and should have their own BTL. The WHO publishes a large volume of health materials in Spanish, and so a Spanish version of the BTL may also be developed. Another long-term strategic plan is to update the content of the BTL.

## The Impact of the Library

While there has not yet been a formal evaluation of the impact of the BTL, BTL training sessions have provided an opportunity for WHO facilitators to determine how appropriate and relevant the project is in different countries. In their written and oral reports, health workers who received training have said that the BTL has helped them to improve the quality of health care in remote areas. For example, they have said that the library helped them in making decisions such as diagnosing and managing diseases. Box 2 shows comments about Senegal's experience in using the library. In addition, the BTL is distributed along with a variety of tools—such as questionnaires for users, managers, and national coordinators—that WHO librarians or information officers use to collect comments and give feedback to the WHO regional offices and to the headquarters in Geneva.

In some countries, national health promotion teams are using the BTL materials for community health education by creating posters and simple brochures in local languages based on the English or French manuals in the library. The WHO Eastern Mediterranean Region (EMRO), which has its headquarters in Cairo, Egypt, and which has already purchased about 250 BTLs, receives the contents of the BTL from the WHO headquarters and “manufactures a wooden Blue Trunk Library equipped with four wheels” [ 7]. It also adds Arabic materials published in the EMRO Region to the original collection. Two other WHO regional offices—in Southeast Asia and the Western Pacific—have purchased BTLs, and we hope to extend the project to the Caribbean and Latin America.

**Box 1. The Online Health Information Revolution**

- The WHO has created its own online bibliographical database, WHOLIS, at http://www.who.int/library/database/index.en.shtml. Other organizations that generate specialized knowledge have launched similar databases.
- The WHO Regional Office for Africa has created AFROLIB, an online library database at http://afrolib.afro.who.int.
- The WHO also leads HINARI, the Health InterNetwork Access to Research Initiative, at http://www.who.int/hinari/en, a program that provides nonprofit institutions in some developing countries with free or very low cost online access to the major journals in biomedical and related social sciences.
- The Global Information Full Text (GIFT) initiative has also been launched for all the WHO offices (it is only accessible to WHO staff). This initiative is a global site license for online access to a targeted selection of the major international databases and full-text journals in the scientific, health, and biomedical fields.
- An increasing number of medical and scientific journals—including those published by Biomed Central ( http://www.biomedcentral.com) and PLoS ( http://www.plos.org)—are adopting an open-access publishing model, in which articles are freely available and readers are licensed to download, distribute, and translate all works.

**Box 2. Impact of the Blue Trunk Library in Senegal**

Marie Khemesse Ngom, at Senegal's Ministry of Health, commented: “Many reasons explain why the Blue Trunk Library is very much used. The users refresh their knowledge after their initial skills acquired in schools. They use this collection in order to read new techniques, standards, and practices in health and medicine. In Kédougou, the Blue Trunk Library manager said that since this collection has been installed in their health district, every year many health workers are passing their professional exams in hygiene and public health. In Nioro, the health district doctor thanks the Blue Trunk Library because he has specialized in Public Health.”

## Abbreviations

BTL: Blue Trunk Library
WHO: World Health Organization
